# Efficacy of Muscle Exercise in Patients with Muscular Dystrophy: A Systematic Review Showing a Missed Opportunity to Improve Outcomes

**DOI:** 10.1371/journal.pone.0065414

**Published:** 2013-06-12

**Authors:** Silvia Gianola, Valentina Pecoraro, Simone Lambiase, Roberto Gatti, Giuseppe Banfi, Lorenzo Moja

**Affiliations:** 1 Clinical Epidemiology Unit, IRCCS Orthopedic Institute Galeazzi, Milan, Italy; 2 Rehabilitation Department, San Raffaele Hospital, Milan, Italy; 3 Laboratory of Movement Analysis, Vita-Salute San Raffaele University, Milan, Italy; 4 IRCCS Orthopedic Institute Galeazzi, Milan, Italy; 5 Department of Biomedical Sciences for Health, University of Milan, Milan, Italy; Johns Hopkins Univ. School of Medicine, United States of America

## Abstract

**Background:**

Although muscular dystrophy causes muscle weakness and muscle loss, the role of exercise in the management of this disease remains controversial.

**Objective:**

The purpose of this systematic review is to evaluate the role of exercise interventions on muscle strength in patients with muscular dystrophy.

**Methods:**

We performed systematic electronic searches in Medline, Embase, Web of Science, Scopus and Pedro as well as a list of reference literature. We included trials assessing muscle exercise in patients with muscular dystrophy. Two reviewers independently abstracted data and appraised risk of bias.

**Results:**

We identified five small (two controlled and three randomized clinical) trials comprising 242 patients and two ongoing randomized controlled trials. We were able to perform two meta-analyses. We found an absence of evidence for a difference in muscle strength (MD 4.18, 95% CIs - 2.03 to 10.39; p = 0.91) and in endurance (MD −0.53, 95% CIs –1.11 to 0.05; p = 0.26). In both, the direction of effects favored muscle exercise.

**Conclusions:**

The first included trial about the efficacy of muscular exercise was published in 1978. Even though some benefits of muscle exercise were consistently reported across studies, the benefits might be due to the small size of studies and other biases. Detrimental effects are still possible. After several decades of research, doctors cannot give advice and patients are, thus, denied basic information. A multi-center randomized trial investigating the strength of muscles, fatigue, and functional limitations is needed.

## Background

Muscular dystrophy is a genetic disorder that gradually weakens the body's muscles [1] limiting person’s functional capacity [2]. It's caused by incorrect or missing genetic information that prevents the body from making the proteins needed to build and maintain healthy muscles [1].

During the last three decades, important progress has been made in the field of muscular dystrophies, leading to the discovery of molecular [3], and genetic [4] causes underlying the disease [5]. There is no cure for muscular dystrophy: scientific advances have not been paralleled by discoveries of effective therapeutic tools so far. Patients have to rely on symptomatic treatments in which continuous physiotherapy is supposed to play a central role [6].

It has been debated for many years whether muscle exercise is beneficial or harmful for patients with myopathic disorders. The role of exercise in the management of these patients remains controversial [6], [7]. Because muscle weakness is the main problem, muscular exercise would be valuable if it helped to counteract the loss of muscle tissue and strength [6]. This theoretical model is established in healthy individuals [8], [9] and in patients with many neurological diseases (e.g. stroke [10], [11], multiple sclerosis [12], etc.). However, hand weakness caused by overwork has been suggested in case reports of patients with facioscapulohumeral muscular dystrophy [13] and scapuloperoneal muscular dystrophy [14], and has also been suggested from animal studies [15], [16]. Given that the evidence for a deleterious effect of strength exercise in patients with muscular dystrophy is largely anecdotal, it is important to conduct a systematic search to point out the effects of muscular exercise in experimental settings.

## Objective

The purpose of this systematic review is to evaluate the role of exercise interventions on muscle strength in patients with muscular dystrophy.

## Methods

### Eligibility Criteria

Trials were included if they met the following criteria: 1) randomized or quasi-randomized controlled trials (RCTs) (namely, controlled trials with inappropriate randomization strategies [17]); 2) inclusion of patients with Duchenne’s muscular dystrophy (DMD), Becker’s muscular dystrophy (BMD), limb-girdle dystrophy (LD), facioscapulohumeral dystrophy (FSHD) and myotonic dystrophy (MyD); 3) muscular exercise was the core of the intervention and was assessed on the basis of muscle strengthening or physical capacity and expressed as a peak torque of strength, motor function or fatigue; and 4) training lasted at least ten weeks. Eligibility was not restricted by language, type of publication, or patients’ age. Trials were irrespective of the type of control, with the caveat to exclude any type of exercise. We included the following categories: no intervention at all, usual care without exercise, and controlateral limb controls (i.e. the parts of the body were randomized to the intervention or control groups). Our approach was ‘inclusive’ so as to obtain a pragmatic overall picture of research in this field.

### Search Strategy

To identify the studies, we searched the following electronic databases: Medline (since 1966), Embase (since 1974), Web of Science (since 1950), Scopus (since 1996) and Pedro (since 1999). The adopted search strategy, which was developed using as key words ‘muscular dystrophy’, ‘physical therapy’, ‘rehabilitation,’ was similar across all databases. We examined the reference list of potentially eligible studies and contacted experts in the field to identify additional trials. The last search was run in June 2012 and it was up to date in February 2013.

### Outcomes

The primary outcome was the peak torque of muscle strength. Secondary outcomes were motor abilities, endurance, and the adverse effect fatigue.

### Study Selection

The literature search was conducted by one investigator (SG). Two researchers (SG and VP) independently screened all studies for eligibility by titles and abstracts. Full texts were then evaluated for inclusion. Disagreements between reviewers were resolved by consensus; if no agreement could be reached, the opinion of a third author (LM) was consulted to be determinant.

### Data Collection

Two authors (SG and VP) independently extracted and entered the data from studies into the data extraction form. Information was extracted from each included trial regarding: (i) characteristics of trial participants (age, sex, type of dystrophy and muscle involved); (ii) characteristics of studies (study design, study year and country where the study was performed); (iii) outcomes (peak torque of muscle strength, motor abilities, endurance and fatigue).

Disagreements were resolved by discussion; if no accord was reached, it was planned that a third author (LM) would decide the matter. Authors were contacted if the reported data were insufficient or unclear.

### Quality Assessment

Two reviewers (SG, VP) independently evaluated the risk of bias in the following domains: study design, randomization, blinding of outcome assessors, reporting of inclusion and exclusion criteria, withdrawals and dropouts, and adverse events. Every domain could be classified as “high” or “low” risk of bias. If the information reported in the paper was not sufficient, the domain was defined as “unclear”. Two reviewers (SG and VP) independently assessed the risk of bias. A third reviewer (LM) was consulted in instances where consensus could not be reached between the two reviewers.

### Statistics

To explore effect modifiers, we pooled studies trough a subgroup analysis according to the type of dystrophy, strengthening exercise, control, and muscle involved. All data were continuous. To quantify the effect associated with each outcome, we used the mean difference (MD) or standardized mean difference (SMD) with a 95% confidence interval (CI) according to the scales measuring the outcome. We assumed that clinical heterogeneity was pervasive in this field, given the potential wide range of patient populations, the types of intervention (e.g. different for intensity or duration), and the types of controls. We summarized data in a meta-analysis using the random effect models described by DerSimonian and Laird [18] according to the inverse variance method. We assessed the presence of heterogeneity utilizing the I-squared statistic (I^2^), which estimates the percentage of variation between study results that is due to heterogeneity rather than sampling error. The I^2^ statistic indicated the percentage of variability due to between-study (or inter-study) variability as opposed to within-study (or intra-study) variability [19]. An I^2^ value greater than 50% was classified as substantially heterogeneous. For the meta-analyses, we used the longest available follow-up data. If no follow-up data were available we used end-of treatment data.

All analyses were done with Review Manager (RevMan5) software version 5.2.

## Results

### Studies Selection

**Figure 1:**
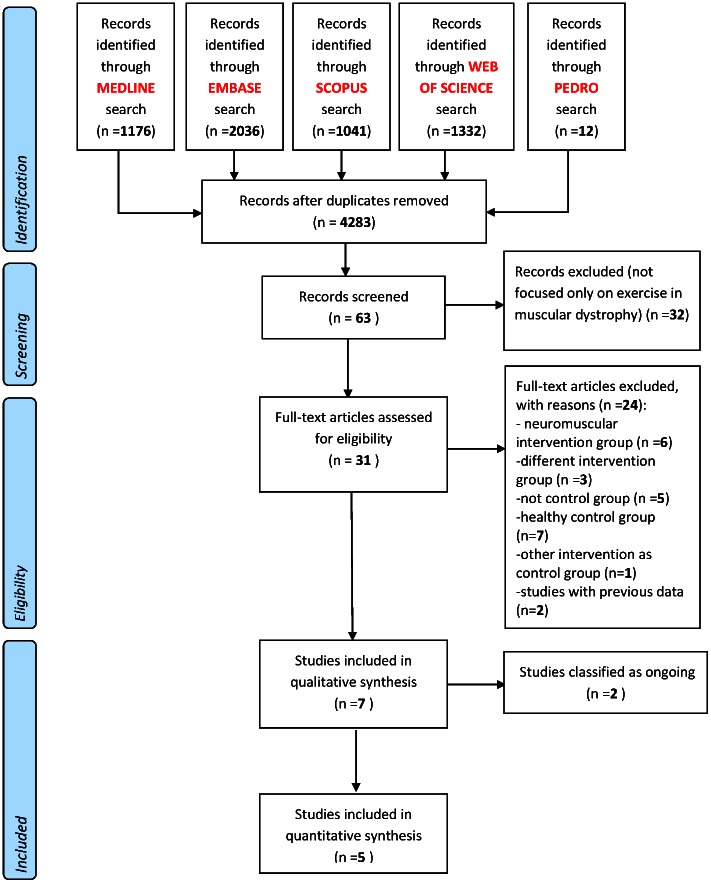
Literature flowchart.

The literature search identified 4283 references. After the exclusion of duplicates and irrelevant references, 58 were left. Thirty-one were eligible for inclusion, their full texts scrutinized for further detail. Twenty-four studies were excluded because: i) they included patients with muscular dystrophy in a wider group of patients with neuromuscular diseases (n = 6) (7–12); ii) used interventions combined with other than exercise interventions (e.g. medication) (n = 3) [20], [21], [22], had no control group (n = 5) [23], [24], [25], [26], [27], had a healthy control group (n = 7) [28], [29], [30], [31], [32], [33] or other interventions as control group (n = 1) [34]. Results from two trials were reported in more than one publications [7], [35]. We counted these as one entry for meta-analyses. Finally, seven trials were included [36], [37], [38], [39], [40], [41], [42], Figure 1.

### Study Characteristics

The included studies are three RCTs [38], [39], [40], two controlled clinical trials [41], [42] and two ongoing RCTs [36], [37]. Main features of the seven trials are summarized in Table 1. Overall, 242 participants were considered, with the number of participants ranging from 4 to 75. The majority of patients (52%) had FSHD, followed by MyD (32%) and DMD (16%). In adult dystrophies, the age of participants ranged from 22 to 48 years. In DMD, the age of participants ranged from 4 to 11 years. All trials were conducted in Western countries between 1978 and 2011.

**Table 1:** Characteristics of studies included.

First author, year	Age (y),mean (SD)	Dystrophytype	Sample size(N)	Muscle involved	Intervention group	Length of intervention	Outcome	Outcome Measures	Design study
De Lateur, 1978	4–11^1^	DMD	4	1) Knee extensor	30 contraction submaximal isokinetic exercise	six-months	1) Muscle strength	1) Maximal peak torque	CT
Jansens, 2010	Not reported	DMD	30	Legs and arms training by bicycle	30-min sessions (15 min leg and 15 min arm training). Intensity: low to moderate	six-months	1) Muscle strength 2) Fatigue 3) Motor function	1) Modified MRC 2) Six-Minute Bicycle Test 3) ICF functional dimensions	RCT ongoing
Kierkegaard, 2011	Training 44 (11), control 41 (15)	MyD type1	35	General involvement	60-min Friskis&Svettis® Open Doors^2^ (60–80% of maximum heart rate)	14 weeks	1) Fatigue 2)Endurance 3) Motor function	1) BORG 2) Six-minute walk test (m) 3) Timed-stands test, timed up-and-go test	RCT
Lindeman, 1995	Training 40 (11), control 37 (10)	MyD	28	1) Knee extensor 2) Knee flexor	1)1–8 weeks:3×25; 60% of 1 RM^3^, 2) 9–6 weeks:3×15; 70% of 1 RM^3^, 3)17–4 weeks:3×10; 80% of 1 RM^3^	24 weeks	1) Muscle strength 2)Endurance 3) Motor function	1) MVIC^4^ Isokinetic torque 2) Test 80% MVC (sec) 3) Descending stairs, climbing stairs, standing up from a chair, standing up from lying supine, walking 6 m(comfortably), walking 50 m (fast) (sec)	RCT
**First author, year**	**Age (y),** **mean (SD)**	**dystrophy** **type**	**Sample size(N)**	**Muscle involved**	**Intervention group**	**Length of intervention**	**Outcome**	**Outcome Measures**	**Design study**
Tollback, 1999	22–48^1^	MyD	5	1) Knee extensor	Free weight, 3×10 repetitions at 80% of 1 RM^3^ (max. repetition)	12 weeks	1) Muscle strength	1) MVIC^4^	CT
Van der Kooi, 2004	Training 36 (9), control 39 (9),	FSHD	65	1) Elbow flexor 2) Ankle dorsiflexor	1) 0–8 weeks^5^∶2×5 to 10 repetitions (10 RM^3^ weights) 2) 9–17 weeks: sets of 8 repetitions (8 RM^3^ weights) 3) From week 18: setsx 5 repetions (5 RM^3^ weights). All exercises: 30 minutes.	52 weeks	1) Muscle strength 2) Fatigue	1) MVIC 2) CIS-fatigue	RCT
Voet, 2010	Not reported	FSHD	75	Aerobic Exercise Training (AET)	Two groups: (1) AET+ usual care, (2) CBT+usual care	16 weeks	1) Fatigue 2) Motor function	1) CIS-fatigue 2) ICF functional dimensions	Multi-center RCT ongoing

1. Range of age in years.

2. 9–10 min warm-up exercises followed by 3–4 min flexibility exercises. Strength exercises for arms, back and abdomen were then done on all fours, and prone and supine, for 6–7 min. This was followed by balance exercises in standing for 3–4 min. Aerobic activities followed for 11–12 minutes, and after 9–10 min cool-down exercises the program was rounded off with stretching and relaxation.

3. Repetition Maximum (RM). An X RM is the weight a person can lift X times at a steady controlled pace through the full range of joint motion.

4. MVIC, maximum voluntary isokinetic strength.

5. Between these two sets of dynamic exercises patients performed a 30-second isometric exercise with the same training weight.

Exercises as an intervention proposed by the authors varied by the following categories: standard strengthening of muscles [38], [39], [41], [42], a comprehensive group of exercises (e.g. aerobic, strengthening, flexibility, balance) supported by music [40], aerobic exercise training (AET) [37], and legs and arms cycling [36]. For the control, five studies used the patient as a unit of allocation as two, the anatomical district (i.e. controlateral limb). All randomized the controls to no training or usual care. The length period of exercise intervention ranged from 12 weeks to 52 weeks. The outcome measures used to assess muscle strength, motor function, fatigue and endure are summarized in Table 1.

### Methodological Quality

**Figure 2:**
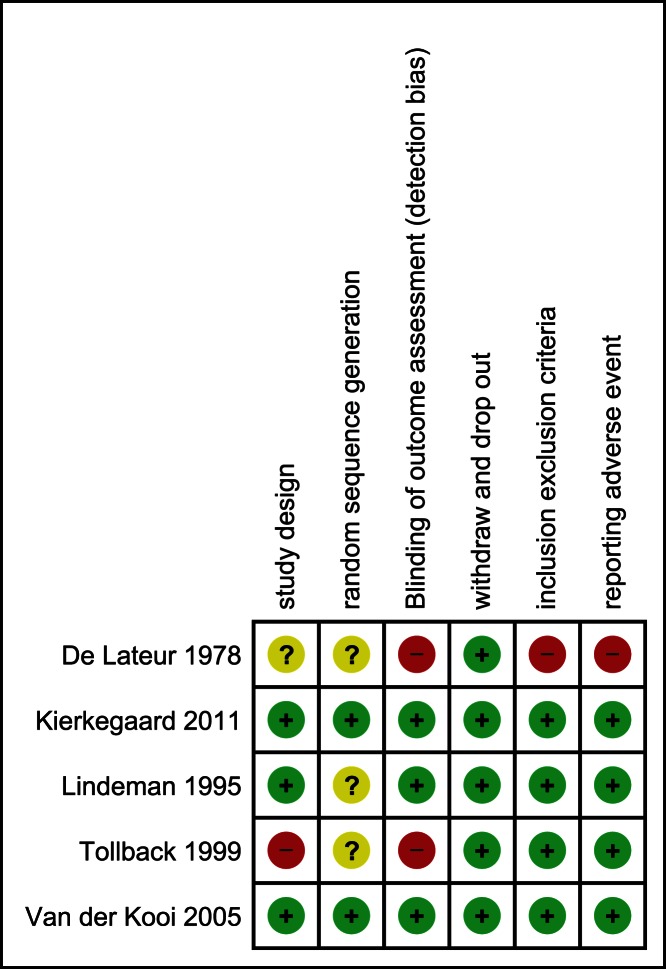
Risk of bias table. Legend: Red (-) = high risk of bias; Yellow (?) =  unknown risk of bias; Green (+) =  low risk of bias.

Risk of bias evaluation is reported in Figure 2. Most of the studies were judged as having a low risk of bias (reported in green). Three of the five studies were randomized and used a blinded procedure [38], [39], [40]. Two of the five studies declared the use of the random sequence generation [38], [40]. All studies reported details of dropouts or withdrawals. Four of the five reported the inclusion and exclusion criteria and reported adverse events [38], [39], [40], [41].

### Quantitative Data Synthesis: Effect of Interventions

#### Muscle strength

Four of the five trials [38], [39], [41], [42] comprising 102 patients measured the maximal voluntary isometric contraction (MVIC) with an isokinetic dynamometer. Three studies [39], [41], [42] evaluated the knee extensor, one [39] considered the knee flexor, and one [38] looked at the effect of training on elbow and ankle flexors.

**Figure 3:**
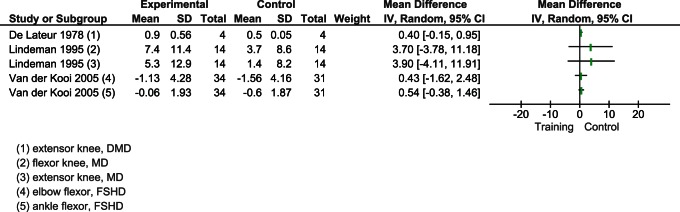
Overall effect of muscular exercise on strength as MVIC.

**Figure 4:**
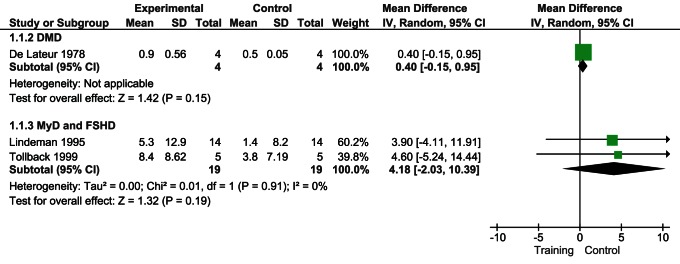
Effect of muscular exercise on strength as MVIC knee extension.

To underline the cumulative trend effect of exercise, we plotted graphically (i.e. no meta-analysis) all effects sizes irrespective of muscle district and dystrophy. In all studies the direction of the effect of muscular exercise is toward a positive but not significant change on muscle strength (Figure 3). We meta-analyzed studies to account for muscle district and dystrophy. Knee extensors were evaluated in DMD [42] and MyD [39], [41]. The effect of muscular exercise was not significantly different (respectively, MD 0.40 CI 95% −0.15 to 0,95; p = 0.15 and MD 4.18 CI 95% −2.03 to 10.39; p = 0.19) (Figure 4).

#### Motor abilities

**Figure 5:**
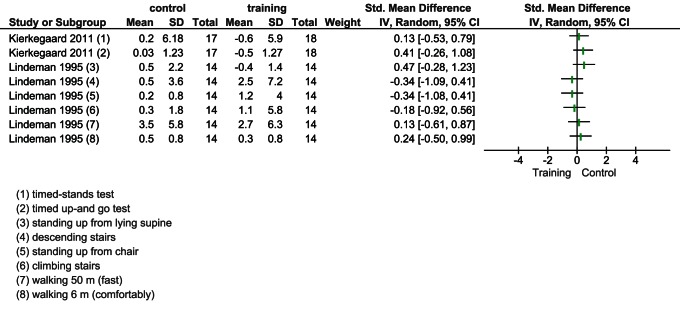
Overall effect of muscular exercise on motor function.

Two studies [39], [40], totaling 63 patients, tested motor abilities using different instruments. Again, both studies were first plotted without pooling data irrespective of activities. Across all activities, there was an absence of evidence of a significant effect of muscular exercise on muscle strength (Figure 5). When we meta-analyzed results, any combination of activities resulted in non-significance differences (results not shown).

#### Endurance

**Figure 6:**
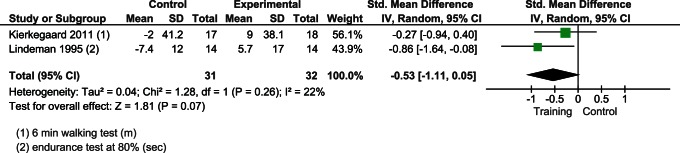
Effect of muscular exercise on endurance.

Two studies [39], [40] considered endurance in 63 patients. The results slightly favored exercise in improving the endurance but the differences were not significant (SMD −0.53; CI 95% from −1.11 to 0.05; p = 0.26) (Figure 6).

#### Adverse effect – fatigue

Two studies provided information about fatigue [38], [40]. The results were heterogeneous, preventing us from meta-analyzing them. Van der Kooi [38] reported that patients did not experience fatigue or muscle soreness. In Kierkegaard [40], the post-walk Borg rating of perceived exertion showed no significant between-group differences after the intervention.

### Ongoing Trials

Jansen et al. are studying the effects of low-intensity physical training on muscle endurance and functional abilities in boys with DMD [36]. In a three-arms trial, Voet et al. are exploring the effect of aerobic exercise training (AET) and cognitive-behavioral therapy (CBT) on the reduction of chronic fatigue in patients with FSHD [37].

## Discussion

Muscular exercise for patients with muscular dystrophy may be useful, not useful, or detrimental. Our analysis does not exclude any of these possibilities: the confidence intervals are so wide that they cover all the possibilities, from ample benefit to possible disadvantage. This might depend on the wide clinical heterogeneity between experimental design and clinical characteristics in the studies, or on the small numbers of patients accumulated in more than 30 years of research [6]. The implication for practice is that exercise can neither be recommended nor excluded from therapies, given their unknown benefit. In a disease still without a cure, the research community was unable to provide any answer of interest to the basic question posed by all patients and their relatives:*“Is muscle exercise beneficial or detrimental in patients with muscular dystrophies?”.*

Only five published and two ongoing trials were retrieved from our extensive search. Although considered as rare diseases, dystrophies do not have a negligible prevalence. Considering the most frequent dystrophy, MyD, there is a conservative prevalence of at least 1 in 8000 people worldwide [43] and a prevalence rate of 2.2–5.5 per 100 000 inhabitants in Western Europe [44]. This is only focusing on one type of dystrophy. A crude estimate of the number of patients diagnosed with muscular dystrophy, who could have been randomized to answer to our question, can be made from this rate. From 1978, when patients were enrolled in the first RCT, until today, 36 900 new patients were diagnosed with MyD in Europe alone. Only 0.6% were randomized each year, one patient for every 174. If we consider patients in USA, Australasia, and the rest of Europe, estimating 1% as the feasible proportion of patients eligible for RCTs, at least 8 750 additional patients would have led to a clearer – and earlier - recognition of the benefits or risks of exercise, thereby potentially shifting the advice given to patients based on theories as well as anecdotic experience and evidence.

Patients with dystrophies are cared for and monitored in few centers. All centers can be easily connected in a network. In this perspective, a multicentre clinical trial is essential. A research infrastructure supporting the trial, such as European Clinical Research Infrastructure Network (ECRIN), might additionally be of great value [45]. Results stratified by age, sex, type and duration of disease, and symptom severity could well be an attainable goal. The pioneering studies with active programs of exercises in dystrophy were done by Abrahamson and Rogoff [24] and by Hoberman [23] in the 1950 s. Their findings on exercise to strengthen muscles in dystrophy, however, are hard to interpret since they did not use un-exercised control patients with comparable disease severity [46]. Controls are important in any trial but essential in muscular dystrophy that often involves very young patients. In this case, three progressing phenomena have to be controlled: 1) physiological muscle volume increase, 2) musculoskeletal growth that produce a load increase, hindering the functional abilities if not accompanied by a contemporary increase of muscle strength and, 3) the degenerative process of muscular dystrophy itself [42]. Major shortcomings of studies about exercise in muscular dystrophy include the lack of appropriate control groups and small sample sizes, often fewer than 35 patients [36], [38], [39], [40], [41], [42]. Finally, we noted scant and variable use of outcome measures such as fatigue and motor abilities.

Studies in this field are not easy and face a number of practical difficulties. First, because of large inter-individual differences, training programs must be adapted to each patient’s physical ability and capacity (28, 34). Clinical care of these patients is complex since they can present multiple severe conditions. This status might be a barrier against their inclusion in standardized experiments [47] and can worry health professionals about potential worsening clinical conditions. Second, non-adherence to an exercise plan is an ever-present threat to the validity and outcome of any intervention study, especially those in which people with muscular dystrophy are involved as cognitive and behavioral problems are associated aspects of the disorder (34). In addition, progressive loss of muscle function is complicated by cardio-respiratory co-morbidity, progressive joint contractures, and deformity. Despite the difficulties of conducting RCTs in this field, the two ongoing RCTs involving 30 DMD patients and 75 FSHD patients [36], [37] are welcome. Although the total sample size is limited, these RCT are of great value and will add to our scarce knowledge.

Patients and clinicians need to make well-informed decisions. The strident mismatch between patient priorities and research agenda has been vibrantly criticized [48]. Several reasons have been proposed as to why research is still so remote from patients’ needs. The research system gives priority to questions that may have limited clinical impact, moving through hypotheses without always completing earlier studies, and assigning low – or no – priority to the publication of negative results. Advocacy groups for muscular dystrophy invest millions to support research, hoping to promote better care [48]. Although they have the responsibility for allocating funding, hypotheses are often driven by research professionals- competing interests, academic and financial, may, therefore, be involved [49]. Liberati (34) proposed that the essential components of any new research governance strategy are: 1) analysis of existing and ongoing research, 2) including patients when setting the research agenda, 3) reducing the role of experts and stakeholders with vested interests, not only financial. The most innovative aspect of the Liberati’s thinking was the call for a formal and substantial commitment by all stakeholders (charities, government, drug companies) to gather and discuss the research agenda. Each funding entity, driven by its selection of priorities from its limited point of view, would, thus, be obliged to place its priorities into a wider perspective, making their choices more transparent and efficient. This intellectual inheritance has become the fulcrum of an international movement - referred as the Liberati initiative - to help those who fund health research to be fully aware of what matters concerning patients, physicians, institutions, and all the other members of society who are involved [50]. The aim is to integrate the experiences and preferences of patients with the pipeline of scientific research so as to promote the acknowledgement of uncertainty about the effect of treatment [51], [52].

This review has some limitations. We included studies with controlateral limb exercise although there are reports that unilateral training enhances strength and performance in the controlateral untested limb [53]. Neural adaptations are possibly responsible for controlateral strength gains [54], [55]. Discarding these studies would have further reduced the information we collected. If there was any bias, the inclusion of these studies would have diluted the effect. It would have been ideal to analyze the findings with and without these studies in a sensitivity analysis. However, given the paucity of patients, we missed this opportunity. Finally, a Cochrane review limited to controlateral limb exercise studies came to similar inconclusive conclusions [56].

### Conclusions

Our meta-analysis is inconclusive. Only five RCTs addressed the question of whether muscular exercise improves muscle strength in a dystrophic population compared to no intervention. Exercise might be useful, not useful, or even detrimental. Although it is accepted that exercise has a positive role in many diseases, we cannot generalize this finding to muscular dystrophy. Even though the effects were consistent across studies, the benefit was limited in size and might have been due to small-study effect or other biases. Detrimental effects also remain a possibility. Practice recommendations regarding the prescription of exercise should stress the uncertainty surrounding the utility of muscle exercise. Patients today must decide whether to start or continue exercise on the basis of expert opinions or anecdotic experience at best. There is simply no evidence about the type, frequency, and intensity of exercise. RCTs targeting this question are urgently needed. A new research governance strategy for neuromuscular disorders should be adopted, beginning by concentrating efforts and optimizing funding research through few multi-center trials that will answer questions that matter to patients. The strength of muscles, fatigue and functional limitations, as well as pain need to be considered in the next generation of RCTs.

## Supporting Information

Checklist S1 (DOC)
